# D-mannose suppresses the angiogenesis and progression of colorectal cancer: D-mannose suppresses the angiogenesis of colorectal cancer

**DOI:** 10.3724/abbs.2025043

**Published:** 2025-04-21

**Authors:** Yu Du, Xinchao Zhang, Yixin Xu, Yuefan Zhou, Yanping Xu

**Affiliations:** 1 Nourse Centre for Pet Nutrition Wuhu 241200 China; 2 Tongji Hospital School of Life Sciences and Technology Tongji University Shanghai 200120 China

**Keywords:** D-mannose, VEGFR2, GSK3β, TFE3, CRC, lysosome

## Abstract

Angiogenesis is an important factor influencing the development of solid tumors, and vascular endothelial growth factor receptor-2 (VEGFR2) is a central regulator of angiogenesis. Antibodies and inhibitors against VEGFR2 have been widely used in various malignancies. However, the regulatory mechanism of VEGFR2 has not been fully clarified. Here, we show that D-mannose can significantly inhibit angiogenesis and tumor growth by degrading VEGFR2. Specifically, D-mannose inactivates GSK3β by promoting the phosphorylation of GSK3β at Ser9, enhances the nuclear translocation of TFE3, and promotes lysosomal biogenesis, thereby increasing the lysosome-mediated degradation of VEGFR2. Thus, D-mannose significantly inhibits the proliferation, migration, and capillary formation of human umbilical vein endothelial cells (HUVECs) *in vitro*. Oral administration of D-mannose dramatically inhibits angiogenesis and tumor growth in mice. Our findings reveal a previously unrecognized anti-tumor mechanism of D-mannose by destabilizing VEGFR2 and provide a new strategy for the clinical treatment of colorectal cancer (CRC).

## Introduction

Colorectal cancer (CRC) is a common malignant tumor of the gastrointestinal tract. Recent statistics have shown that the incidence of CRC is the third highest overall incidence and the second highest mortality rate [1]. Despite advances in screening procedures and adjuvant therapy, the incidence, prevalence and mortality of CRC remain high [1]. Therefore, the development of new treatments and drugs for CRC is urgently needed.

Angiogenesis plays a crucial role in the growth, recurrence and metastasis of CRC [2]. The formation of new blood vessels provides nutrients and oxygen for tumors, thus promoting the rapid proliferation of cancer cells [3]. Various growth factors, cytokines, and mechanical cues are implicated in the induction of angiogenesis within tumors [4]. The main pro-angiogenic drivers are vascular endothelial growth factor (VEGF) and its receptors VEGFR1–3 [5], among which VEGFR2 has been identified as a critical component of VEGF-mediated angiogenesis [6].

VEGFR2 is a large 151 kDa membrane protein consisting of 7 extracellular immunoglobulin (Ig)-like domains, a single transmembrane helix, and a split intracellular kinase domain [7]. VEGFR2 has been implicated in angiogenesis in many solid tumors, including breast cancer, colon cancer, hepatoma, and gastric cancer [ 2, 8– 10]. VEGF induces receptor dimerization and subsequent phosphorylation by binding to VEGFR2, thereby maintaining downstream signaling. Phosphorylation of VEGFR2 promotes downstream activation of ERK1/2, AKT, and eNOS, which are critical for human umbilical vein endothelial cell (HUVEC) proliferation, migration, and angiogenesis [11]. Therefore, VEGFR2-blocking therapies that reduce its activity or stability and downstream signaling are of great clinical importance.

Lysosomes consist of an acidic lumen and a lysosomal membrane formed by a phospholipid bilayer and are responsible for degrading a variety of biomolecules, including proteins, lipids, carbohydrates and nucleic acids [12]. A number of studies have shown that the functional state and spatial distribution of lysosomes are closely related to tumor angiogenesis [ 13, 14]. It has been shown that Myosin 1b promotes tumor angiogenesis in CRC by inhibiting the degradation of HIF-1α by the autophagic lysosomal pathway and increasing VEGF secretion [15]. D-mannose is a six-carbon sugar, a C-2 epimer of glucose, which enters mammalian cells via the same transporters as glucose [16]. When D-mannose enters the cell, it can be phosphorylated by hexokinase (HK) and produce mannose-6-phosphate (M6P), which can either enter the glycolytic pathway directly or provide a substrate for glycosylation [16]. A number of studies have shown that D-mannose enhances the sensitivity of cancer cells to chemotherapy, radiotherapy, and immunotherapy [ 17, 18] and that D-mannose ameliorates ulcerative colitis in mice by inhibiting macrophage activation through the inhibition of IL-1-β production [19]. Recently, D-mannose was found to inhibit oxidative reactions and block phagocytosis in experimental neuroinflammation [20]. However, the role of D-mannose in angiogenesis and in the treatment of CRC is unclear.

In this study, we found that D-mannose significantly decreased the protein level of VEGFR2 and effectively inhibited angiogenesis and tumor growth in CRC. Mechanistically, D-mannose promotes VEGFR2 degradation by enhancing TFE3-mediated lysosomal biogenesis. Importantly, oral administration of D-mannose strongly inhibited angiogenesis and tumor growth in CRC in mice. Overall, D-mannose may be useful for the clinical treatment of CRC.

## Materials and Methods

### Cell culture and reagents

Human colon cancer HCT116 cells (Meilunbio, Dalian, China) and HUVECs (kindly provided by Dr. Lei Lv of Fudan University, Shanghai, China) were cultured in DMEM (Meilunbio) supplemented with 10% FBS (Gibco, Carlsbad, USA) and 100 μg/mL penicillin and streptomycin. The human renal clear cell adenocarcinoma line 786-O cells (Meilunbio) were cultured in RPMI-1640 (Meilunbio) supplemented with 10% FBS and 100 μg/mL penicillin and streptomycin. The cells were cultured in an environment containing 5% CO _2_ at 37°C. The drugs used in this study included D-mannose (3458-28-4; Sigma-Aldrich, St Louis, USA), L-mannose (L82450; Acmec, Shanghai, China), fructose (A100226; Sangon Biotech, Shanghai, China), galactose (A600215; Sangon Biotech), fucose (A606132; Sangon Biotech), MG132 (HY-13259; MCE, Shanghai, China), NH _4_Cl (A100621; Sangon Biotech), 3-MA (T1879; TagertMol, Boston, USA), cycloheximide (CHX, S7418; Selleck, Houston, USA), rapamycin (MB1197; Meilunbio), VEGF (HY-P73556; MCE), 2-deoxy-D-glucose (2-DG, A602241; Sangon Biotech), and indirubin-3′-monoxime (I3M, T5200; TagertMol).

### siRNA transfection

After transient transfection with siRNAs, including siRNAs against the control, *MITF*, *TFE3* and *TFEB* (GenePharma, Shanghai, China) using Lipofectamine RNAiMAX Transfection Reagent (13778-150; Invitrogen, Carlsbad, USA), HUVECs were cultured in serum-free DMEM for 4‒6 h. Then the medium was replaced by DMEM complete culture medium (Meilunbio) supplemented with 10% FBS and 100 μg/mL penicillin and streptomycin. The siRNAs used were designed as previously reported [21], and the sequences are as follows: siRNA control (siNC): 5′-UUCUCCGAACGUGUCACGUTT-3′; siMITF#1: 5′-GGUGAAUCGGAUCAUCAAG-3′; siTFE3#1: 5′-AUCCCUGCUCUCUUCAGUGTT-3′; siTFEB#1: 5′-UGUAAUGCAUGACAGCCUG-3′). Another set of siRNAs, including siMITF#2 (sc-35934), and siTFE3#2 (sc-38507), and siTFEB#2 (sc-38509), was purchased from Santa Cruz (Santa Cruz, USA).

### Western blot analysis and co-immunoprecipitation (Co-IP)

Cells were treated with mannose, inhibitor of proteasome degradation MG132, inhibitors of lysosomal acidification NH _4_Cl, inhibitor of the autophagy pathway that inhibits the formation of autophagosomes 3-MA, the protein synthesis inhibitor CHX with indicated time and then the total proteins from the cells were extracted via RIPA lysis buffer with 1% PMSF (Beyotime, Shanghai, China). The proteins were separated by 1% SDS-PAGE and transferred onto a nitrocellulose membrane (Cytiva, Shanghai, China). The membrane was blocked with 5% skim milk at room temperature for 1.5 h, and then incubated with primary antibody overnight at 4°C. After being washed with TBST three times, the membrane was incubated with the secondary antibody at room temperature for 1 h. Then, it was washed with TBST again three times and visualized with the Chemiluminescent HRP Substrate (Millipore, Darmstadt, Germany) on the Chemiluminescent Imaging System (Tanon, Shanghai, China). For Co-IP, the cell lysate supernatants were incubated with anti-FLAG beads for 4‒6 h at 4°C. The beads were washed 3 times with 0.3% NP-40 buffer, heated with 5× Native Gel Sample Loading Buffer and subjected to western blot analysis. All the antibodies used are commercially available and are shown in Supplementary Table S1.

### Quantitative real-time PCR (qRT-PCR)

Total RNA was extracted from cells via the EZ-press RNA Purification Kit (EZBioscience, Roseville, USA), and RNA was synthesized into cDNA via a gDNA remover mixed with a reverse transcription master mix (EZBioscience). qRT-PCR was performed on an Applied Biosystems 7300 plus Sequence Detection System (Applied Biosystems, Foster City, USA). The Ct values were analyzed via the 2 ^–ΔΔCt^ method, and the final results are presented as relative fold changes. The mRNA expression of genes was normalized to the expression of the *β-actin* gene. The sequences of primers used in this study are listed in Supplementary Table S2.

### Separation of nuclear and cytoplasmic proteins

The separation of nuclear and cytoplasmic proteins was conducted via a nuclear protein and cytoplasmic protein extraction kit (P0027; Beyotime) according to the manufacturer’s instructions. One percent phase phosphatase inhibitor and 1% protease inhibitor were added when reagents A and B were used. Western blot analysis was used to examine the acquired protein samples.

### LysoTracker Red staining

The cells were incubated at 37°C for 60 min in serum-free medium supplemented with Lysosomal Tracking Red DND-99 dye (50 nM, 40739ES50; Yeason, Shanghai, China) for specific staining of lysosomes. The cell nuclei were then labeled with Hoechst dye (Beyotime). After the samples were rinsed with PBS, they were observed, and photographs were taken via a CKX53 fluorescence microscope (Olympus, Tokyo, Japan).

### N-acetyl-β-D-glucosidase (NAG) activity assay

The cells were washed with PBS, extracts were added, and the cells were scraped off with a cell scraper. The cells were disrupted by ultrasonication and then centrifuged to remove the supernatant. The NAG assay was performed using a corresponding kit (BC4290; Solarbio, Beijing, China). Finally, the absorbance at 400 nm was measured.

### Colony formation assay

The cells were seeded at 1000 cells/well in a 6-well plate. The medium was changed every three days, and the culture was stopped after two weeks when visible colonies formed. After being washed with PBS, the cells were fixed with 4% methanol for 20 min and stained with 0.1% crystal violet (Beyotime) for 30 min. The crystal violet was discarded, and the cells were washed with PBS and photographed using a BX53 microscope (Olympus).

### Wound healing assay

When the cell density reached 90%–100%, the cells were scratched with a sterile pipette tip. The medium was removed, the cells were washed with PBS three times to remove floating cells, and then the cells were treated with D-mannose and VEGF for 24 h. Images were captured under the BX53 microscope at 0 and 24 h, and the migration rate was measured via ImageJ software.

### Cell migration assay

Cell migration was evaluated using a 24-well Transwell plate with polycarbonate membrane inserts (Corning Co., Corning, USA). A homogenous single-cell suspension (200 μL, 5 × 10 ^4^ cells/well) was added to the upper chambers with serum-free medium, and 500 μL of complete medium was added to the lower chambers. After 24 h of incubation in a CO _2_ incubator at 37°C, the migrated cells were fixed with methanol and stained with crystal violet for 30 min. Images of different visual fields were captured under the BX53 microscope, the number of cells was counted.

### Tube formation assay

After 50 μL of Matrigel (Corning, New York, USA) was spread per well, the 96-well plate was placed in a humidified incubator at 37°C for 30 min to allow the Matrigel to cure. Well-grown HUVECs were resuspended with the appropriate drugs to make a cell suspension of 1 × 10 ^5^ cells/mL. The prepared cells were added to the 96-well plate coated with Matrigel (200 μL per well) and cultured. The number of tubes formed was counted under the BX53 microscope.

### Animal experiments

The animal experiments in this study conformed to the ethical review approved by the Department of Experimental Animal Science of Tongji University (Approval No. TJAB09624103). The experiment was conducted as previously reported [22]. Briefly, male nude BALB/c mice (6–8 weeks old) were purchased from GemPharmatech Laboratory Animals (Nanjing, China), and assigned into 2 groups with 6 mice in each group. The mice were fed under SPF conditions and were allowed free access to food and water. The light/dark cycle was conducted every 12 h, and the temperature was controlled (22–23°C). CT-26 cells were kindly provided by Dr. Lei Lv (Fudan University). CT26 cells (1 × 10 ^6^) were injected into the right backs of the mice. D-mannose was added to the drinking water at 10%, and 200 μL of 20% D-mannose solution was administered to the mice by gavage every three days. At the end of the experiment, the tumor volume and weight were recorded, and the tumor volume was calculated via the following formula: tumor volume = length/2 × width ^2^. Tumor tissues were collected for subsequent western blot and immunohistochemical analyses.

### Immunohistochemical (IHC) analysis

The obtained tissues were fixed with 4% paraformaldehyde, after which the tissues were embedded in paraffin and sectioned. After dewaxing the tissue sections, antigen retrieval was performed via citrate (A100529; Sangon Biotech) buffer at a concentration of 10 mM, pH 6.0, at 100°C. The sections were washed with PBS, and the tissue was circled with an immunohistochemical pen to create a hydrophobic isolation zone. The sections were blocked with 3% BSA (Millipore) for 1 h. Overnight incubation was performed with a primary antibody, and after washing, incubation was performed for 30 min at room temperature with a secondary antibody. The sections were then stained with 3,3′-diaminobenzidine (DAB) and counterstained with hematoxylin. The sections were subsequently observed and photographed under the BX53 microscope and analyzed via ImageJ software. All the antibodies used for IHC are commercially available and are shown in Supplementary Table S1.

### Statistical analysis

Graphpad Prism 8.0.2 software (GraphPad Software, San Diego, USA) was used to statistically analyze the experimental data. Two independent samples *t*-tests were used for comparisons between two groups, and one-way analysis of variance (ANOVA) was used for comparisons among multiple groups. *P*  < 0.05 was considered statistically significant.

## Results

### D-mannose significantly decreases VEGFR2 protein level

**Figure 1:**
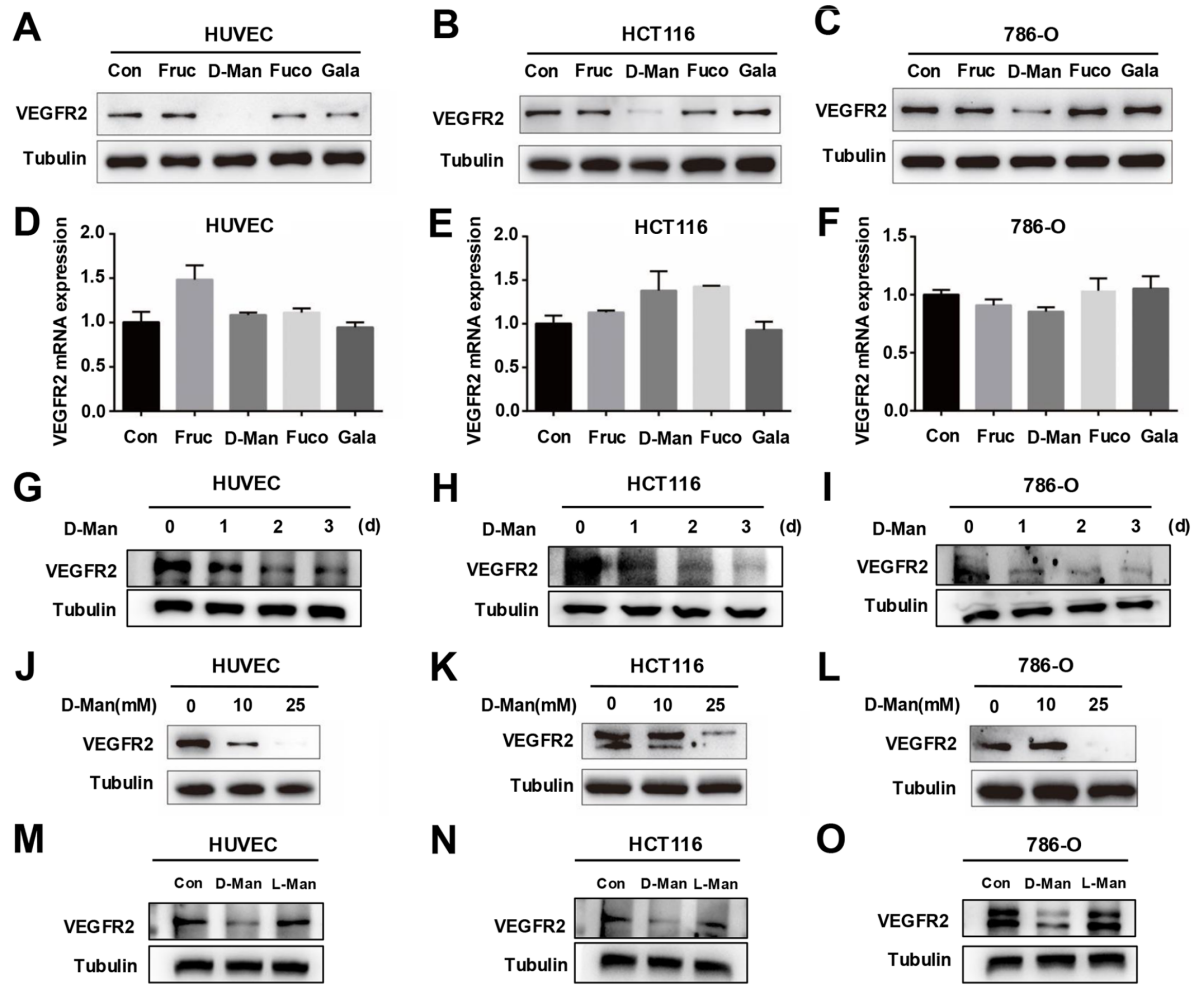
Figure 1 VEGFR2 protein level is downregulated under D-mannose treatment (A–C) Western blot analysis of VEGFR2 protein level in HUVECs (A), HCT116 cells (B), and 786-O cells (C) after treatment with or without different hexoses for 48 h. (D–F) qRT-PCR analysis of VEGFR2 mRNA level in HUVECs (D), HCT116 cells (E), and 786-O cells (F) treated with or without different hexoses for 48 h. Data are presented as the mean ± SD from 3 independent experiments. (G–I) Western blot analysis of VEGFR2 protein level in HUVEC (G), HCT116 cells (H), and 786-O cells (I) treated with 25 mM D-mannose for different durations. (J–L) Western blot analysis of VEGFR2 protein level in HUVEC (J), HCT116 (K), and 786-O (L) cells treated with different concentrations of D-mannose for 48 h. (M–O) Western blot analysis of VEGFR2 protein level in HUVEC (M), HCT116 (N), and 786-O (O) cells treated with D-mannose (25 mM) or L-mannose (25 mM) for 48 h.

VEGFR2 is critical for angiogenesis and tumor development [23]. To explore whether metabolites affect the expression level of VEGFR2, we evaluated the effects of different hexoses on VEGFR2 in human umbilical vein endothelial cells (HUVEC) and the colon cancer cell line HCT116. We observed that D-mannose, but not other hexoses, significantly reduced VEGFR2 protein level in both cell lines ( Figure 1A,B). A similar phenomenon was observed in the human renal clear cell adenocarcinoma line 786-O ( Figure 1C). In addition, we examined the changes in VEGFR2 at the transcriptional level and found that the above hexoses did not affect the mRNA level of *VEGFR2* ( Figure 1D–F). We next treated the cells with D-mannose for different durations and observed a time-dependent decrease in VEGFR2 protein level ( Figure 1G–I). Furthermore, D-mannose treatment reduced VEGFR2 protein level in a dose-dependent manner ( Figure 1J–L). To clarify whether the effect of D-mannose on VEGFR2 is isoform specific, we treated cells with D-mannose or L-mannose and found that only D-mannose significantly reduced the protein level of VEGFR2 ( Figure 1M–O), which suggests that D-mannose has an isoform-specific regulatory effect on the VEGFR2 protein level. Since D-mannose can be phosphorylated by HK to produce M6P, which subsequently enters the glycolytic pathway, we further investigated whether the downregulation of VEGFR2 level by D-mannose is due to D-mannose or its metabolites. We used the HK inhibitor 2-DG to inhibit the phosphorylation of D-mannose to form M6P, thereby preventing it from entering glycolysis. Compared with D-mannose treatment alone, D-mannose still downregulated VEGFR2 protein level in the presence of 2-DG, and even slightly more VEGFR2 was downregulated ( Supplementary Figure S1A–C). These results suggest that D-mannose rather than its metabolites downregulates VEGFR2 protein level.

### D-mannose mediates VEGFR2 degradation via the lysosomal pathway

**Figure 2:**
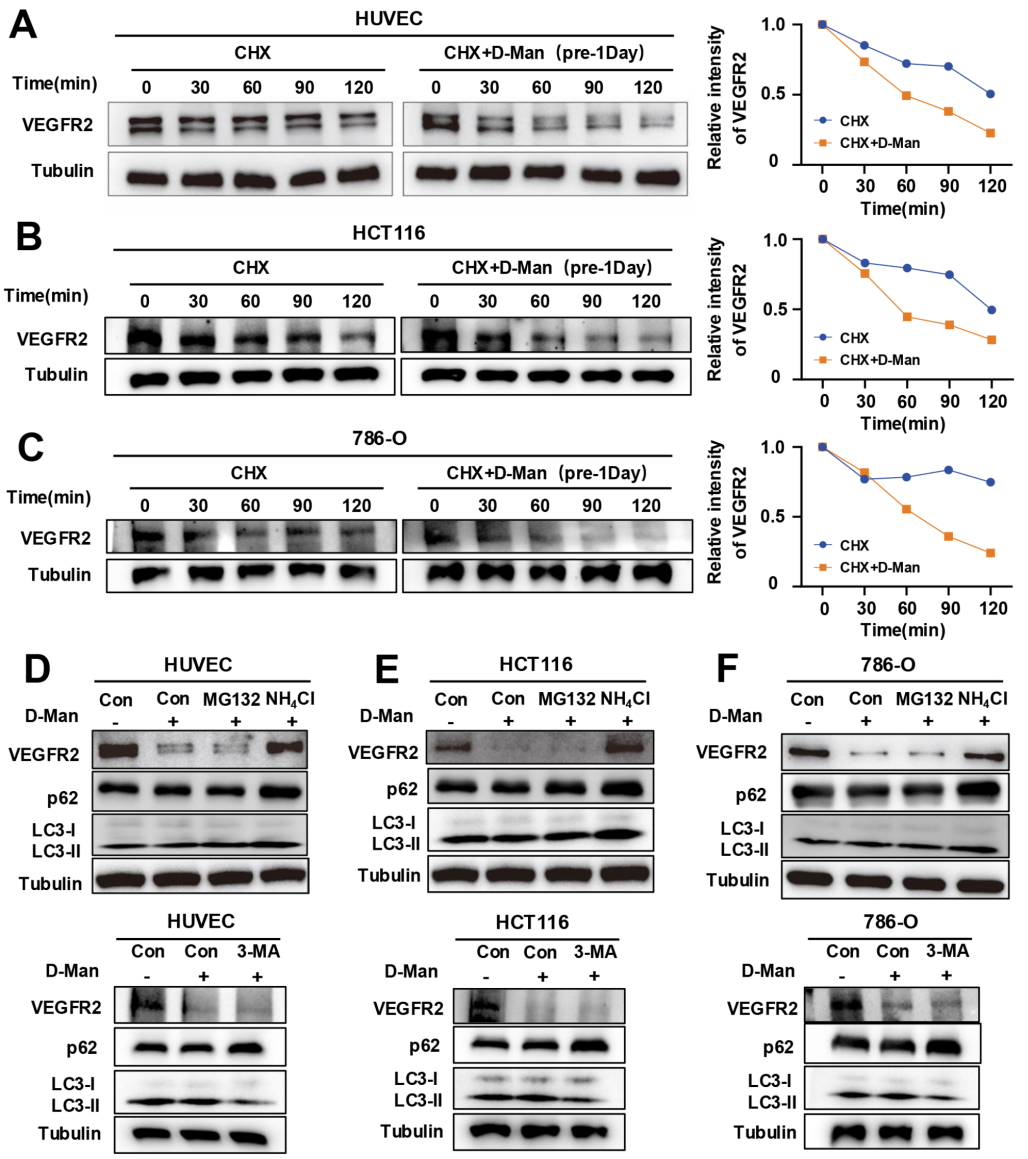
Figure 2 The lysosomal pathway contributes to D-mannose-mediated downregulation of VEGFR2 (A–C) The half-life of VEGFR2 under D-mannose (25 mM) treatment was determined via CHX (100 μg/mL)-chase assay in HUVECs (A), HCT116 cells (B), and 786-O cells (C). ((D–F)) Western blot analysis of VEGFR2, p62 and LC3 levels in HUVECs (D), HCT116 cells (E), and 786-O cells (F) treated with D-mannose (25 mM, 48 h) in the absence or presence of NH4Cl (50 mM, 24 h), MG132 (10 μM, 6 h) or 3-MA (5 mM, 24 h).

Next, we aimed to determine whether D-mannose reduces the expression of VEGFR2 by affecting its stability. We treated cells with the protein synthesis inhibitor CHX and found that VEGFR2 was degraded faster in cells treated with D-mannose than in control cells ( Figure 2A–C), indicating that D-mannose reduced the protein stability of VEGFR2. Given that the proteins in eukaryotes are degraded mainly via the proteasome [24] or lysosomal [25] pathway, we then investigated the potential contribution of the proteasome or lysosomal pathway to D-mannose-induced degradation of the VEGFR2 protein. We treated cells with NH _4_Cl (an inhibitor of lysosome acidification), MG132 (an inhibitor of proteasome degradation) and 3-MA (an inhibitor of the autophagy pathway that inhibits the formation of autophagosomes, inhibits the conversion of LC3-I to LC3-II, and increases p62 accumulation) in the presence of D-mannose. Interestingly, only NH _4_Cl partially restored D-mannose-induced degradation of VEGFR2 but not MG132 or 3-MA ( Figure 2D–F). The data indicate that D-mannose promotes VEGFR2 degradation via lysosomes.

### D-mannose increases the biogenesis of lysosomes and promotes TFE3-dependent degradation of VEGFR2

**Figure 3:**
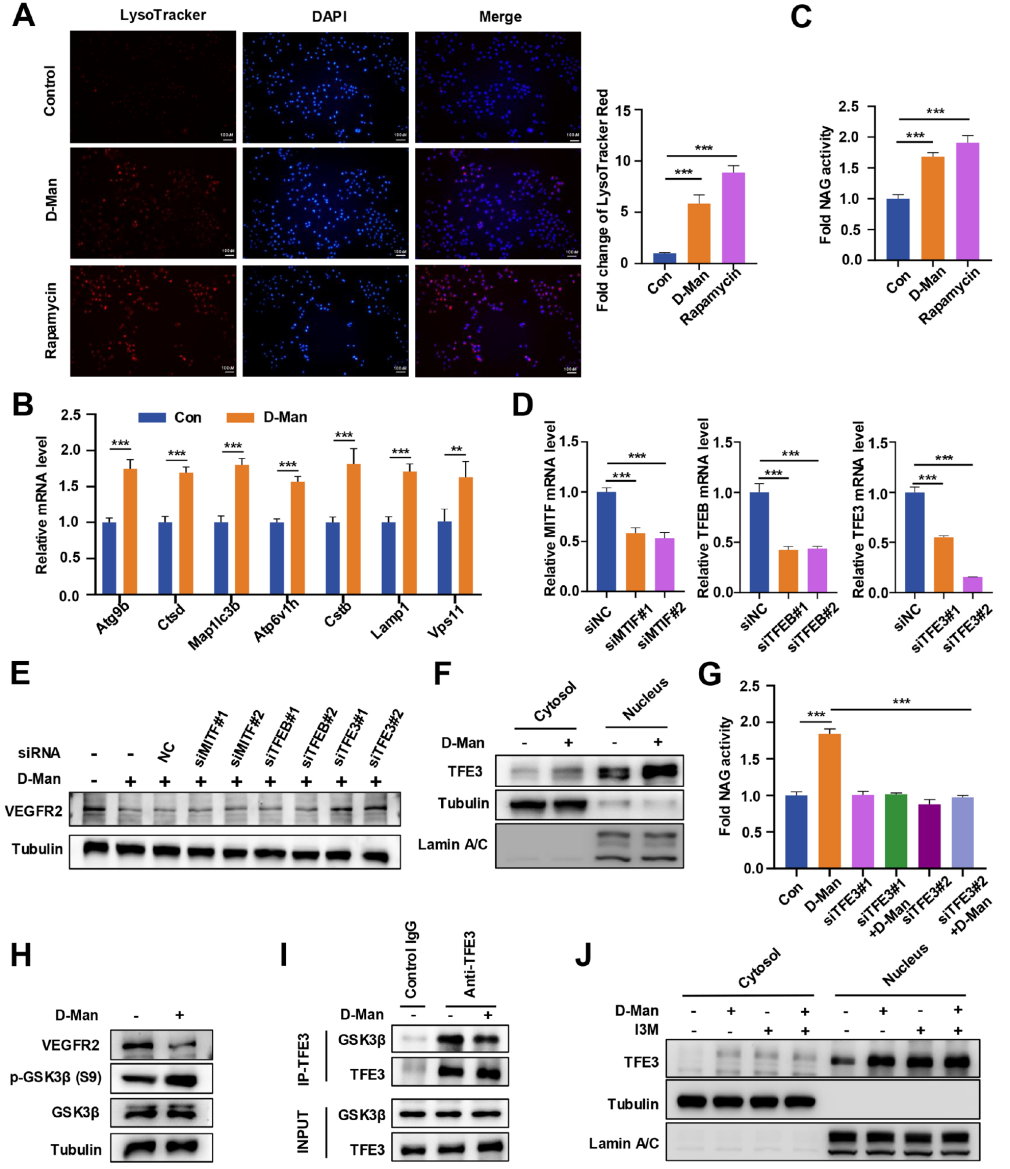
Figure 3 D-mannose promotes VEGFR2 degradation by enhancing TFE3-mediated lysosomal biogenesis (A) LysoTracker Red staining of HUVEC treated with or without D-mannose (25 mM) or rapamycin (10 nM) for 24 h (scale bar = 100 μm). Hoechst was used to label the nuclei. The quantification of the LysoTracker intensity is shown in the right panel. ***P < 0.001. (B) qRT-PCR analysis of HUVEC treated with or without D-mannose (25 mM, 48 h). Data are presented as the mean ± SD from 3 independent experiments. **P < 0.01, ***P < 0.001. (C) The relative lysosomal NAG activity of D-mannose- or rapamycin-treated HUVECs was examined. Data are presented as the mean ± SD from 3 independent experiments. ***P < 0.001. (D) qRT-PCR analysis of the efficiency of siRNAs targeting different MiT/TFE family genes. Data are presented as the mean ± SD from 3 independent experiments. ***P < 0.001. (E) Western blot analysis of the VEGFR2 level in D-mannose (25 mM, 48 h)-treated HUVECs transfected with siRNAs targeting the negative control (NC), MITF, TFE3 or TFEB. (F) Western blot analysis of the cytosolic and nuclear locations of TFE3 in HUVECs treated with D-mannose (25 mM, 24 h). (G) Relative lysosomal NAG activity in HUVECs transfected with control or TFE3 siRNAs after D-mannose treatment (25 mM, 24 h) was determined. ***P < 0.001. (H) Western blot analysis of the levels of p-GSK3β (S9), GSK3β and VEGFR2 in HUVECs treated with D-mannose (25 mM) for 24 h. (I) Co-IP analysis of the interaction between TFE3 and GSK3β in HUVECs treated with or without D-mannose (25 mM, 24 h). (J) Western blot analysis of the cytosolic and nuclear locations of TFE3 in HUVECs treated with D-mannose (25 mM, 24 h) or I3M (10 μM, 4 h) alone or in combination.

To further explore how D-mannose promotes the lysosomal degradation of VEGFR2, we first examined whether D-mannose treatment affects lysosomal activity by using LysoTracker Red. Interestingly, similar to rapamycin, D-mannose significantly increased lysosomal activity ( Figure 3A). In addition, D-mannose significantly upregulated the expressions of many lysosomal biogenesis-related genes, including *Atg9b*, *Map1lc3*, and *Vps11* ( Figure 3B). We next examined NAG activity under D-mannose treatment and found that D-mannose was able to increase NAG activity ( Figure 3C), suggesting that D-mannose can increase lysosomal protease activity. Lysosomal biogenesis is triggered primarily by the MiT/TFE family, which includes TFEB, TFE3, and MITF, thereby increasing the number of lysosomes and promoting protein degradation [ 21, 26]. We knocked down *TFEB*, *TFE3*, and *MITF* by siRNA in HUVECs and found that D-mannose-induced VEGFR2 degradation was significantly reversed when *TFE3* was silenced but not when *TFEB* or *MITF* was silenced ( Figure 3D,E), suggesting that D-mannose-induced VEGFR2 degradation is dependent on TFE3. Consistently, D-mannose treatment promoted TFE3 expression and increased its nuclear translocation ( Figure 3F). In addition, knockdown of *TFE3* abolished the increase in NAG enzyme activity induced by D-mannose ( Figure 3G). In conclusion, the above results indicate that D-mannose promotes the nuclear translocation of TFE3 and lysosomal biogenesis to degrade VEGFR2.

Next, we explored the molecular mechanism by which D-mannose promotes TFE3 nuclear translocation. It was previously reported that glycogen synthase kinase 3 beta (GSK3β)-mediated phosphorylation of TFE3 promotes its subsequent nuclear export and inactivation [ 27, 28]. Western blot analysis revealed that D-mannose treatment significantly increased the phosphorylation of GSK3β at Ser9 in HUVEC and inhibited the binding of GSK3β to TFE3 ( Figure 3H,I), suggesting that D-mannose inhibits the activity of GSK3β and its interaction with TFE3, thereby increasing the nuclear translocation of TFE3. In addition, TFE3 is regulated by a variety of kinases, including mTOR and GSK3β [29]. To confirm that D-mannose regulates TFE3 exclusively through GSK3β, we examined the effects of treatment with D-mannose alone, the GSK3β inhibitor (I3M) alone, or both on the nuclear translocation of TFE3 to assess the activity of TFE3 in HUVEC. We detected similar level of TFE3 nuclear translocation in all three cases, suggesting that D-mannose regulates TFE3 exclusively through GSK3β ( Figure 3J). Taken together, these data suggest that D-mannose inactivates GSK3β by inducing its phosphorylation (Ser9), which promotes the nuclear translocation of TFE3, thereby increasing lysosomal biogenesis and subsequent VEGFR2 degradation.

### D-mannose inhibits the proliferation, migration and capillary tube formation of HUVEC *in vitro*

**Figure 4:**
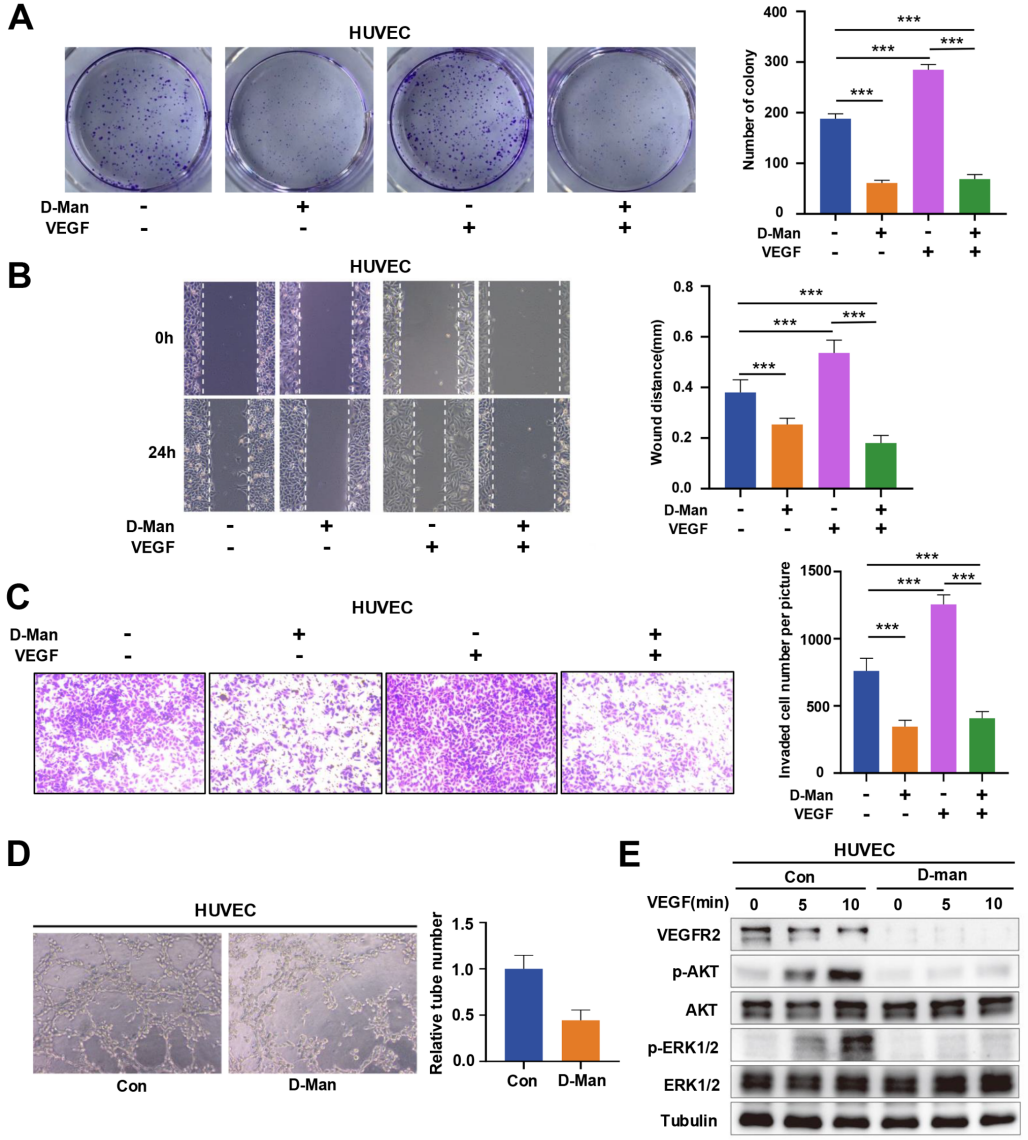
Figure 4 D-mannose inhibits the proliferation, migration and capillary tube formation of HUVECs *in vitro* (A) The proliferation of HUVECs treated with D-mannose (25 mM) or VEGF (50 ng/mL) as indicated was determined via a colony formation assay. ***P < 0.001. (B) Wound healing assay was used to evaluate the effects of D-mannose (25 mM) or VEGF (50 ng/mL) on the migratory ability of HUVECs. ***P < 0.001. (C) Transwell migration assay was used to evaluate the effects of D-mannose (25 mM) or VEGF (50 ng/mL) on the migratory ability of HUVECs. ***P < 0.001. (D) Tube formation of HUVECs in the absence or presence of D-mannose (25 mM) for 24 h. (E) Western blot analysis of the levels of VEGFR2, p-AKT, AKT, p-ERK1/2, and ERK1/2 in HUVECs treated with or without D-mannose (25 mM) for 24 h followed by VEGF (50 ng/mL) treatment for different durations.

VEGF is a key angiogenic growth factor that stimulates proliferation, migration, and capillary tube formation in HUVEC, primarily through VEGFR2 [30]. Therefore, we further examined the effects of D-mannose on the proliferation, migration and capillary tube formation of HUVEC. Through these experiments, we found that D-mannose significantly inhibited VEGF-enhanced cell proliferation ( Figure 4A). In addition, both wound healing and transwell migration assays demonstrated that D-mannose was able to repress the migration of HUVEC ( Figure 4B,C). Moreover, we found that D-mannose dramatically suppressed tube formation via a capillary tube formation assay ( Figure 4D). We also investigated the role of D-mannose in VEGFR2 signaling in HUVEC. The results showed that D-mannose reduced VEGF-induced p-AKT and p-ERK1/2 levels without affecting their protein levels ( Figure 4E). Collectively, these results suggest that D-mannose can inhibit the proliferation, migration and capillary tube formation of HUVEC by suppressing VEGFR2 signaling.

### D-mannose significantly suppresses the growth and angiogenesis of xenograft tumors *in vivo*

**Figure 5:**
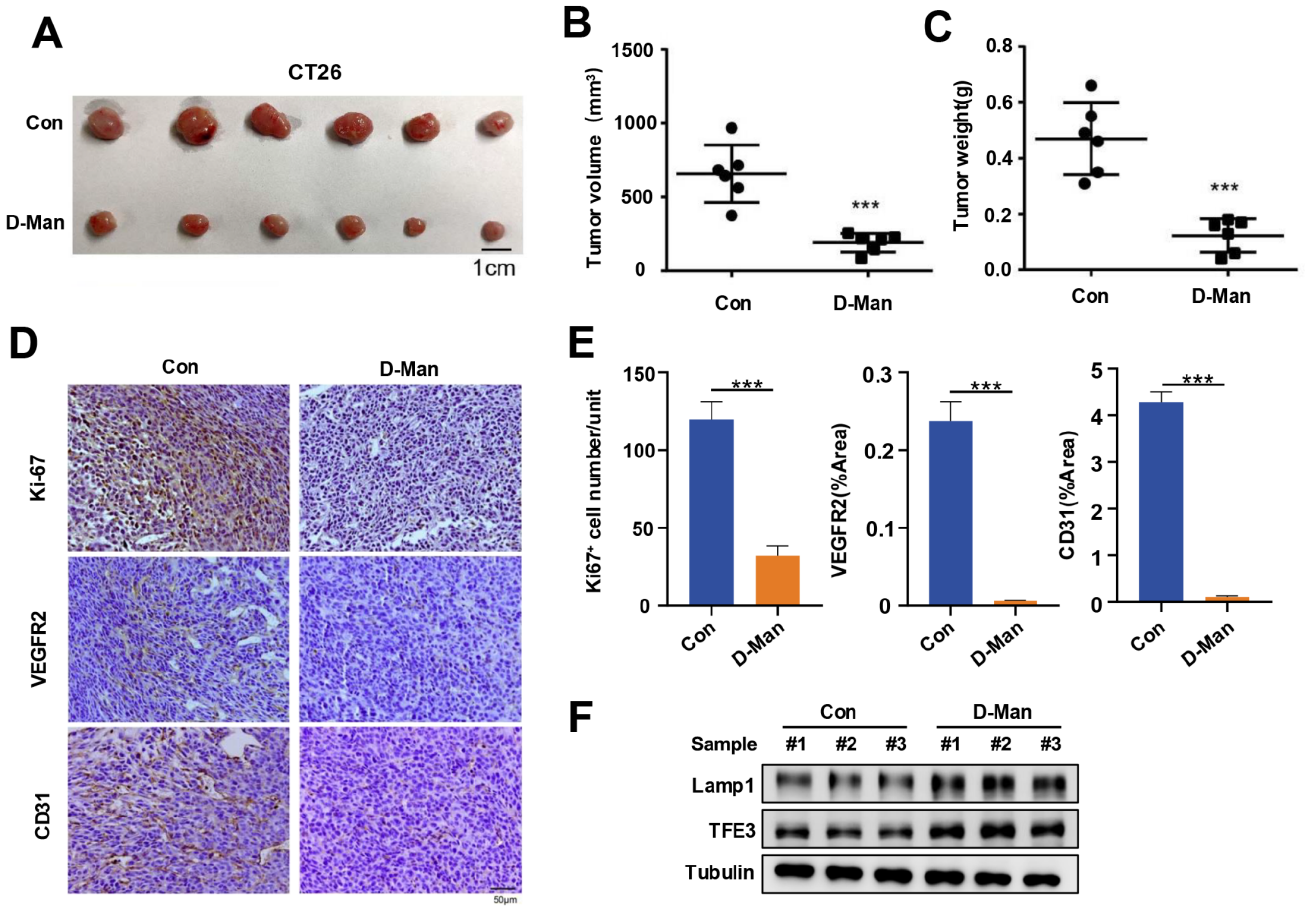
Figure 5 D-mannose significantly inhibits the growth and angiogenesis of xenograft tumors *in vivo* (A) Tumors from nude BALB/c mice treated with or without D-mannose are shown. Representative tumors resected from each group of mice that received different treatments as indicated. n = 6 mice per group. (B) The volume of tumors in each group of mice that received different treatments as indicated was analyzed. ***P < 0.001. (C) The weights of tumors resected from each group of mice that received different treatments as indicated were analyzed. ***P < 0.001. (D) Immunohistochemistry showing Ki67, VEGFR2 and CD31 expressions in tumor tissues as indicated (scale bar = 50 μm). (E) Quantification of the images in (D). Data are presented as the mean ± SD from 6 independent samples from each group. Statistical differences were determined via ordinary one-way ANOVA. ***P < 0.001. (F) Western blot analysis of the protein levels of Lamp1 and TFE3 in tumor tissues.

Since our data revealed that D-mannose inhibited VEGFR2-mediated angiogenesis *in vitro*, we then evaluated whether D-mannose could inhibit the growth of CRC tumors by suppressing tumor angiogenesis *in vivo*. We established a mouse CT26 colon cancer model to clarify the effect of D-mannose on CRC *in vivo*. The results showed that the oral administration of D-mannose reduced tumor growth and tumor weight in the mice ( Figure 5A–C). In addition, IHC staining of tumor tissues revealed that D-mannose also reduced the protein levels of Ki67, VEGFR2, and the vascular marker CD31 ( Figure 5D,E). In addition, we examined the protein levels of TFE3 and Lamp1 (the lysosomal biogenesis-related gene) in tumor tissues via western blot analysis. Consistent with the findings at the cellular level, the oral administration of D-mannose increased the protein levels of TFE3 and Lamp1 in tumor tissues ( Figure 5F). Together, these results suggest that D-mannose suppresses tumor angiogenesis by promoting lysosomal biogenesis, thereby suppressing tumor growth in CRC.

## Discussion

**Figure 6:**
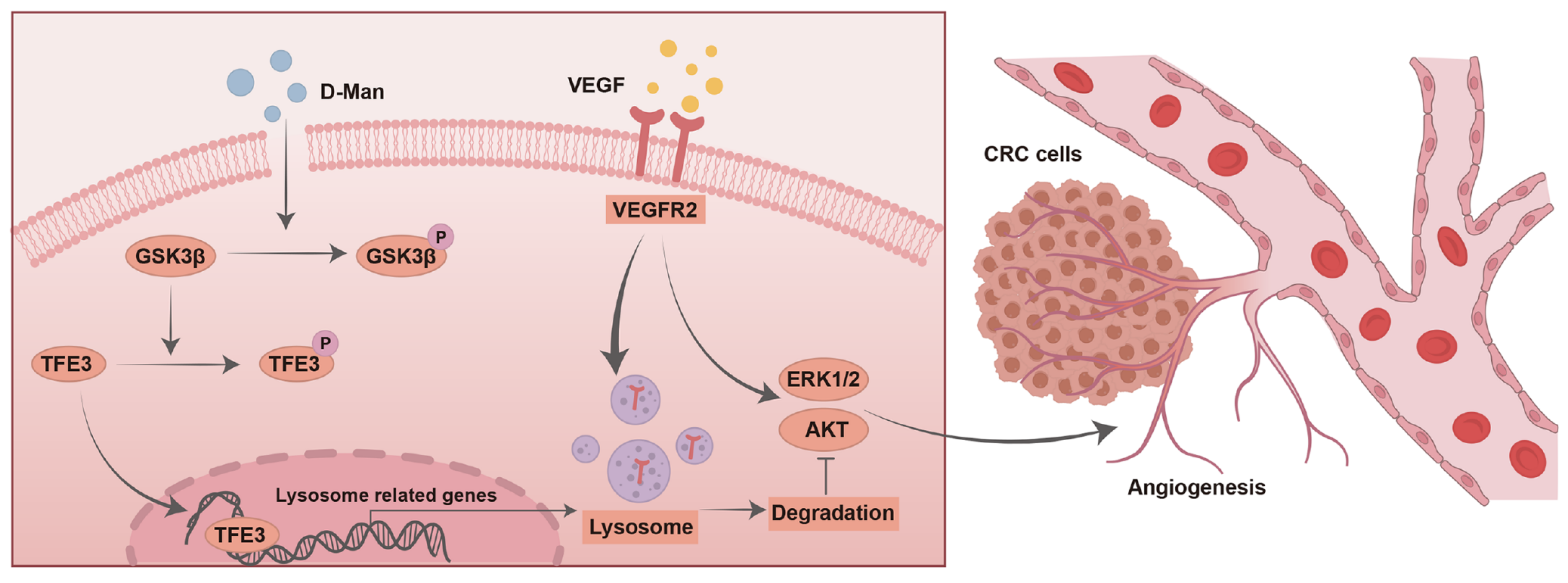
Figure 6 Schematic diagram shows the proposed model of the study D-mannose inactivates GSK3β by promoting the phosphorylation of GSK3β at Ser9, which promotes the translocation of TFE3 to the nucleus, enhances lysosomal biogenesis and induces VEGFR2 protein degradation, thereby inhibiting angiogenesis and tumor growth in CRC.

Many studies have shown that glucose, a major source of cellular energy, plays a crucial role in tumor immunity and therapy. D-mannose, an isomer of glucose, exists naturally in many fruits, yet its function and use in tumor therapy remain largely unexplored. In this study, we explored an unknown mechanism of D-mannose in anti-tumor treatment. We found that D-mannose promotes the degradation of VEGFR2, a major mediator of angiogenic signaling. Further studies revealed that D-mannose inactivates GSK3β, enhances the nuclear translocation of TFE3, upregulates the transcription levels of lysosomal biogenesis-related genes, and promotes lysosomal-mediated degradation of VEGFR2, thereby inhibiting angiogenesis and tumor growth in CRC ( Figure 6).

Angiogenesis, the formation and maintenance of vascular structures in preexisting blood vessels, plays an important role in physiological and pathological conditions such as diabetes, inflammation, and cancer [4]. VEGFR2 is a central regulator of angiogenesis and can be activated in response to VEGF stimulation, thereby promoting downstream activation of ERK1/2 and AKT to induce angiogenic responses [11]. Currently, anti-angiogenic therapy is widely used to treat various cancers. For example, apatinib, an oral receptor tyrosine kinase inhibitor that selectively targets VEGFR2, can inhibit the downstream signaling of VEGFR2 and has good anti-tumor effects on a variety of tumors, including CRC [ 8, 31, 32]. In the present study, we found that D-mannose can significantly downregulate the protein level of VEGFR2, suggesting that D-mannose may be used to treat CRC by inhibiting angiogenesis and is thus a potential option for the treatment of CRC.

The stability and clearance of VEGFR2 are regulated by proteasome or endocytosis/lysosome-dependent degradation [ 33, 34]. In this study, we found that D-mannose promotes the lysosomal degradation of VEGFR2 by increasing lysosomal biogenesis. Lysosomes are acidic compartments containing a variety of different types of hydrolases and are sealed by the plasma membrane, which mediate the degradation of extracellular particles and intracellular components [35]. The increase in lysosomal biogenesis promotes the clearance of accumulated proteins and damaged cells [36]. Lysosomal biogenesis is triggered mainly by the MiT/TFE family of transcription factors, including TFEB, TFE3, and MITF, which can bind to the Coordinated Lysosomal Expression and Regulation (CLEAR) element to stimulate the expressions of its downstream target genes and promote lysosomal biogenesis and protein degradation [ 21, 26]. Our study revealed that D-mannose induces TFE3, but not TFEB or MITF, which is dependent on lysosomal biogenesis in HUVEC. D-mannose treatment efficiently induced the nuclear translocation of TFE3 and increased lysosomal NAG activity, facilitating the degradation of VEGFR2. We further explored the upstream molecular basis of D-mannose-induced TFE3 nuclear translocation and found that GSK3β is involved in this process. D-mannose is able to induce phosphorylation and inactivation of GSK3β, which promotes the nuclear translocation of TFE3.

To adapt to the expansion and growth of new blood vessels, HUVEC need to proliferate, migrate, and invade the basement membrane in response to angiogenic cues [37]. In this study, we found that D-mannose inhibited HUVEC proliferation, migration, and capillary formation. The activation of VEGFR2 promotes the proliferation, migration and activation of the core angiogenic factors ERK1/2, AKT and eNOS [11]. Our study also demonstrated that D-mannose can block the activation of ERK1/2 and AKT downstream of VEGFR2, thereby inhibiting angiogenesis.

In conclusion, our studies suggest that D-mannose treatment results in the inactivation of GSK3β, which enhances the nuclear translocation of TFE3, thereby enhancing lysosomal biogenesis and the degradation of VEGFR2 through lysosomes. Thus, our study reveals a novel mechanism by which D-mannose controls TFE3-mediated activation of lysosomal function and VEGFR2 degradation to inhibit angiogenesis and tumor growth in CRC, providing an experimental basis for the potential application of D-mannose in the clinical treatment of CRC.

## Supporting information

656FigS1-TabS1-2

## Supplementary Data

Supplementary data is available at *Acta Biochimica et Biophysica Sinica* online.
