# Supplementary material for: D-mannose suppresses the angiogenesis and progression of colorectal cancer: D-mannose suppresses the angiogenesis of colorectal cancer
Source: Acta Biochim Biophys Sin (Shanghai). 2025 Apr 21;57(8):1270–80. doi: 10.3724/abbs.2025043 (PMC12368525; doi:10.3724/abbs.2025043)
Supplement: 656FigS1-TabS1-2 [file 656FigS1-TabS1-2.pdf]

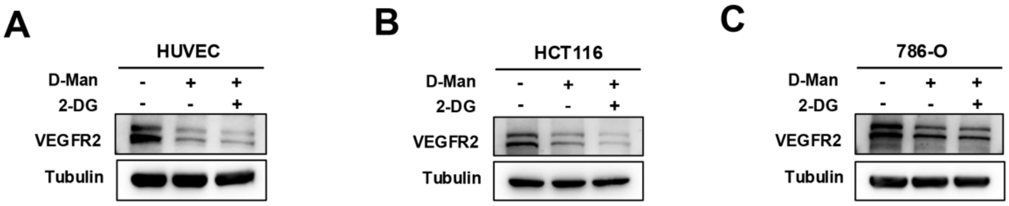

Supplementary Figure S1. VEGFR2 protein level is downregulated under D-mannose treatment (A) Western blot analysis of VEGFR2 protein levels in HUVEC (A), HCT116 (B) and 786-O (C) cells treated with D-mannose (25 mM, 48 h) or D-mannose (25 mM, 48 h) in combination with 2-DG (10 mM, 24 h).

Supplementary Table S1. Antibodies used for western blot analysis and IHC

Antibody	Source	Category No.
Tubulin	Proteintech	66031–1-Ig
VEGFR2	Cell Signaling Technology	9698
VEGFR2	Proteintech	26415-1-AP
Lamin A/C	ABclonal	A19524
TFE3	Proteintech	14480-1-AP
Ki67	HuaBio	HA721115
GSK3β	Proteintech	22104-1-AP
p-GSK3β (S9)	Proteintech	67558-1-Ig
CD31	HuaBio	ER31219
Lamp1	Proteintech	21997-1-AP
P62	HuaBio	HA721171
LC3	Proteintech	81004-1-RR

Supplementary Table S2. Sequences of primers used for qPCR

Gene	Forward primer (5′-3′)	Reverse primer (5′-3′)
β-actin	ACCACACCTTCTACAATGAGC	GATAGCACAGCCTGGATAGC
Atg9b	ACCTCCTCCTCCTCCTTCAT	GTGGGAGGGGAAAATGAGGA
Ctsd	AACTGCTGGACATCGCTTGCT	CATTCTTCACGTAGGTGCTGGA
Map1lc3b	CGCACCTTCGAACAAAGAGT	AGCTGCTTCTCACCCTTGTA
Atp6v1h	GGAAGTGTCAGATGATCCCA	CCGTTTGCCTCGTGGATAAT
Ctsb	AGTGGAGAATGGCACACCCTA	AAGAAGCCATTGTCACCCCA
Lamp1	ACGTTACAGCGTCCAGCTCAT	TCTTTGGAGCTCGCATTGG
Vps11	CGGCGCTTCGTTTTCTTCG	CCCGTAGTTTGTAGGCTTGGAA
VEGFR2	CGGTCAACAAAGTCGGGAGA	CAGTGCACCACAAAGACACG
MITF	TACAGTCACTACCAGGTGCAG	CCATCAAGCCCAAAATTTCTT
TFEB	GCAGCCACCTGAACGTGTA	TGTTAGCTCTCGCTTCTGAGT
TFE3	CCGTGTTCGTGCTGTTGGA	CTCGTAGAAGCTGTCAGGAT
